# Antibody response in elderly vaccinated four times with an mRNA anti-COVID-19 vaccine

**DOI:** 10.1038/s41598-023-41399-5

**Published:** 2023-08-29

**Authors:** Alexander Rouvinski, Ahuva Friedman, Saveliy Kirillov, Jordan Hannink Attal, Sujata Kumari, Jamal Fahoum, Reuven Wiener, Sophie Magen, Yevgeni Plotkin, Daniel Chemtob, Herve Bercovier

**Affiliations:** 1 Department of Microbiology and Molecular Genetics, Faculty of Medicine, Hebrew University of Jerusalem, Jerusalem, Israel; 2 Department of General Biology and Genomics, L.N. Gumilyov Eurasian National University, Astana, Kazakhstan; 3 Braun School of Public Health and Community Medicine, Faculty of Medicine, Hebrew University of Jerusalem, Jerusalem, Israel; 4 Department of Tuberculosis and AIDS, State of Israel Ministry of Health, Jerusalem, Israel; 5 Department of Biochemistry and Molecular Biology, Faculty of Medicine, Hebrew University of Jerusalem, Jerusalem, Israel; 6 Department of Clinical Biochemistry, Shaare Zedek Medical Center, Jerusalem, Israel; 7 Department of Anesthesiology, Critical Care and Pain Medicine, Hadassah Medical Center and Faculty of Medicine, Hebrew University of Jerusalem, Jerusalem, Israel

**Keywords:** Medical research, Microbiology, Clinical microbiology, Vaccines, Virology

## Abstract

The humoral response after the fourth dose of a mRNA vaccine against COVID-19 has not been adequately described in elderly recipients, particularly those not exposed previously to SARS-CoV-2. Serum anti-RBD IgG levels (Abbott SARS-CoV-2 *IgG* II *Quant* assay) and neutralizing capacities (spike SARS-CoV-2 pseudovirus Wuhan and Omicron BA.1 variant) were measured after the third and fourth doses of a COVID-19 mRNA vaccine among 46 elderly residents (median age 85 years [IQR 81; 89]) of an assisted living facility. Among participants never infected by SARS-CoV-2, the mean serum IgG levels against RBD (2025 BAU/ml), 99 days after the fourth vaccine, was as high as 76 days after the third vaccine (1987 BAU/ml), and significantly higher (p = 0.030) when the latter were corrected for elapsed time. Neutralizing antibody levels against the historical Wuhan strain were significantly higher (Mean 1046 vs 1573; p = 0.002) and broader (against Omicron) (Mean 170 vs 375; p = 0.018), following the fourth vaccine. The six individuals with an Omicron breakthrough infection mounted strong immune responses for anti-RBD and neutralizing antibodies against the Omicron variant indicating that the fourth vaccine dose did not prevent a specific adaptation of the immune response. These findings point out the value of continued vaccine boosting in the elderly population

## Introduction

Current literature indicates that the fourth dose of mRNA anti-COVID-19 vaccine (the second booster) in adults resulted in a slight increase of the anti-RBD IgG antibodies and of the neutralizing antibody (nAb) levels when compared to the third vaccine (first booster)^1^. These tepid results, performed in a study population devoid of elderly people, raised doubts as to the usefulness of an additional vaccine dose following the first booster^2–4^. Nevertheless, several natural retrospective studies^5–9^ and a randomized clinical trial^10^ on vaccine efficacy (VE) showed that a fourth vaccination (second booster) was in fact beneficial, especially for preventing hospitalization among the elderly including during the Omicron period. However, these studies rarely included the measurement of specific IgG antibody levels or nAb levels, especially in older populations. In addition, the precise contribution of mRNA vaccines to the immune response among vaccine recipients may have been biased due to unnoticed exposure to the virus^11^. In 2022, few individuals can be considered as never having been infected by one or more SARS CoV-2 variants, particularly those in long-term care and assisted living facilities^12^.

The State of Israel Ministry of Health (MoH) approved the administration of the BNT162b2 vaccine in December 2020 (two doses, 21 days apart), a booster (approved in July 2021), and a fourth BNT162b2 dose to protect the elderly during the Omicron (BA.1) wave on December 31, 2021.

This study addresses the immune response to the third and fourth doses of a COVID-19 mRNA vaccine in an elderly population residing in an assisted living facility, where COVID-19 cases were not reported among residents until January 1st 2022. This unique population and setting facilitate the evaluation of the humoral response to vaccine boosters of Elderly never infected by SARS-CoV-2.

## Results

This study followed 46 residents with different levels of frailty^13–15^ (Table 1) who were vaccinated the same day for each dose within 10 days of the MoH recommendations. A third dose (August 2021) was administered 203 days post the first of two doses, administered January 2021, and a fourth dose (January 2022) was administered 150 days post third dose (Supplementary Fig. S1). The residents were vaccinated solely with the monovalent mRNA BioNtech-Pfizer vaccine.

**Table 1:** Characteristics of study participants, by COVID-19 infection status.

		Participants never infected		Participants infected COVID-19	
		N	%	N	%
Participants	46	40	87.0	6	13.0
Age (years)	Median [Q1–Q3]	86 [81–90]		82 [80–89]	
Sex	Female	34	85.0	6	100
	Male	6	15.0	0	0
Comorbidities	DM	3	7.5	1	16.7
	HTN	22	55.0	2	33.3
	Smoking	10	25.0	1	16.7
	Obesity	8	20.0	1	16.7
COVID-19 vaccination doses	Four doses	40	100	6	100
Days from vaccine dose and blood sample [median, IQR]	2^nd^ Dose	195 [194–195]		194 [194–194]	
	3^rd^ Dose	76 [70–81]		76 [70–81]	
	4^th^ Dose	99 [99–135]		139 [99–143]	
Frailty score					
Fit	0–1	29	72.5	4	66.7
Mild	2–3	8	20.0	2	33.3
Moderate	4–5	3	7.5	0	

Median [IQR = Q1;Q3], Q1 is first quarter and Q3 is third quarter; DM is diabetes mellitus; HTN is high blood pressure; Frailty score were calculated according to the literature^13–15^.

Weekly COVID-19 RT-PCR or antigen tests were performed on all the residents in an enhanced “Elder Shield program” under the supervision of the MoH^16^ and 40 out of 46 residents were consistently found negative until May 2022. Likewise, no symptomatic pathologies evoking COVID-19 were reported by these forty residents or by their medical staff. Six residents, who were vaccinated four times, contracted COVID-19 between January 2022 and April 2022, one the week before the fourth vaccine dose and five between 32 to 121 days after the vaccination (Supplementary Table S1).

The median age of the residents was 85 [Q1–Q3: 81; 89] and is consistent among those never infected with SARS-CoV-2 and those who were ultimately infected (Table 1 and Supplementary Table S1). Among all study participants, 87% were female. All study participants who experienced breakthrough cases of COVID-19 were female. All study participants performed all vaccinations at a regular time (Table 1). None of the patients had renal failure. Further details regarding the study population’s characteristics can be found in Table 1 and Supplementary Table S1.

Antibody responses (anti-RBD IgG and nAb tests) were evaluated in blood samples taken one week before the third vaccine (first week of August 2021) (Supplementary Fig. S1, Supplementary Table S1); a second blood sample (October 2021) was taken 76 days median [Q1–Q3: 70; 81] after the third vaccine and a third blood sample (April–May 2022) was drawn 99 days median [Q1–Q3: 99; 135] after the fourth vaccine dose.

### Serum anti-RBD IgG levels

**Figure 1:**
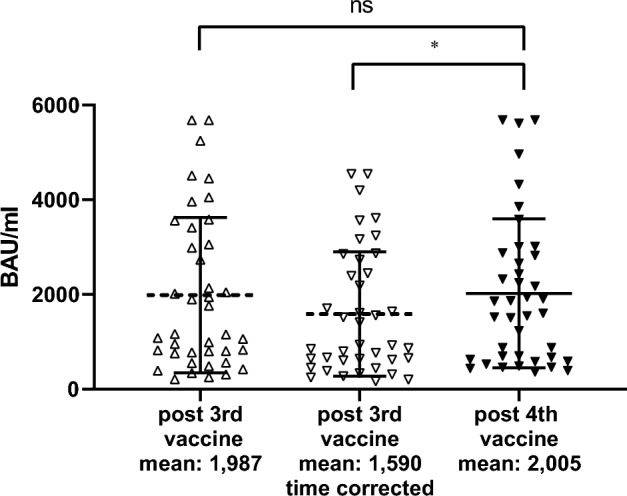
Anti-RBD IgG response in uninfected residents. Anti-RBD IgG were measured in samples taken 76 and 99 days (median) after third (post third vaccine) and fourth (post fourth vaccine) vaccines, respectively, by Chemiluminescent Microparticle ImmunoAssay (CMIA) SARS-CoV-2 IgG II Quant (Abbott, IL, USA), expressed as binding antibody units (BAU) per ml. Each triangle represents one individual. There was initially no significant difference in mean antibody levels between blood sample taken after the third and the fourth vaccines. After time correction of values post third vaccine (20% lower), the increase between third and fourth vaccines became significant. Mean values are indicated below the x-axis. Bars represent 95%CI around the mean. ns: p = 0.8587; *p = 0.0301.

The first blood sample (Supplementary Table S1) obtained the week before the first boost (third vaccine dose) and around seven months after the first vaccine course (two doses, three weeks apart) showed that anti-RBD IgG levels were low (median 50 BAU/mL [Q1–Q3: 24; 126]) but 76 days (median) after the third vaccine dose, the median level increased by a factor of 21 (Table 2). At a median time of 99 days after the second booster (fourth dose of vaccine), the median anti-RBD IgG level increased by 1.5-fold compared to the titers achieved after the third vaccine dose. The geometric mean values were not statistically different in the two time points (Table 2, Fig. 1).

**Table 2:** Anti-RBD IgG levels and neutralizing antibodies in vaccinated and vaccinated breakthrough infections.

		Post third dose		Post fourth Dose		Comparison
		n		n		p-value
Anti-RBD IgG titers BAU/ml						
Non-infected						
Mean (95%CI)		40	1987 (1462; 2512)	40	2025 (1522; 2528)	0.859^a^
Median [Q1-Q3]			1164 [767; 3237]		1730 [651; 2850]	0.547*
Time adjusted mean (95%CI)		40	1590 (1170; 2009)	40	2025 (1522; 2528)	0.030^a^
Breakthrough						
Mean (95%CI)		6	1064 (418; 1711)	6	4,603 (2818; 6389)	0.007^a^
Median [Q1–Q3]			985 [698; 1572]		5,680 [2979; 5680]	0.028*
Neutralizing antibodies titers NT_50_						
Non-infected						
WT	Mean (95%CI)	40	1046 (711; 1382)	40	1573 (1044; 2102)	0.002^a^
	Median [Q1-Q3]	40	669 [333; 1629]	40	1166 [496;1684]	0.001*
BA.1	Mean (95%CI)	40	170 (85; 255)	40	375 (176; 575)	0.018^a^
	Median [Q1-Q3]	40	50 [2; 234]	40	206 [2; 360]	0.001*
Breakthrough						
WT	Mean (95%CI)	6	696 (398; 993)	6	7872 (2435; 13,310)	0.022^a^
	Median [Q1–Q3]		645 [476; 959]		8349 [2036; 13,132]	0.028*
BA.1	Mean (95%CI)	6	9 (2; 28)	6	3538 (2; 7153)	0.053^a^
	Median [Q1–Q3]		2 [2; 2]		3195 [431; 4907]	0.028*

Anti-RBD IgG were measured in samples taken 76 and 99 days (median) after third and fourth vaccines, respectively, by Chemiluminescent Microparticle ImmunoAssay (CMIA) SARS-CoV-2 IgG II Quant (Abbott, IL, USA), expressed as binding antibody units (BAU) per ml. Neutralization capacity [NT_50_] is expressed as a function of reciprocal values of sera dilutions. WT = wild type Wuhan stain; BA.1 = Omicron variant. *Wilcoxon signed ranks test; ^a^Paired samples t-test; Median [Q1;Q3].

Based on the data published by Canetti et al.^3^, Gilboa et al.^17^ and Grassi et al.^18^ showing at least a 0.90% reduction by day of the antibody titers after the third vaccine dose among non-infected vaccinated individuals, here we applied a very conservative ratio of 1.2 for correction and reduced by 20% the values of the IgG titers of the second blood samples obtained 76 days after the first boost (third dose of vaccine) to compare it with the third blood samples obtained 99 days after the second boost (fourth dose of vaccine) (e.g. a difference of 23 days). Using this comparison normalizing the data to 99 days median post vaccination, the mean titer after the second boost was significantly higher than the mean titer after the first boost (p = 0.030, Table 2, Fig. 1).

### Frailty and anti-RBD antibodies levels

**Figure 2:**
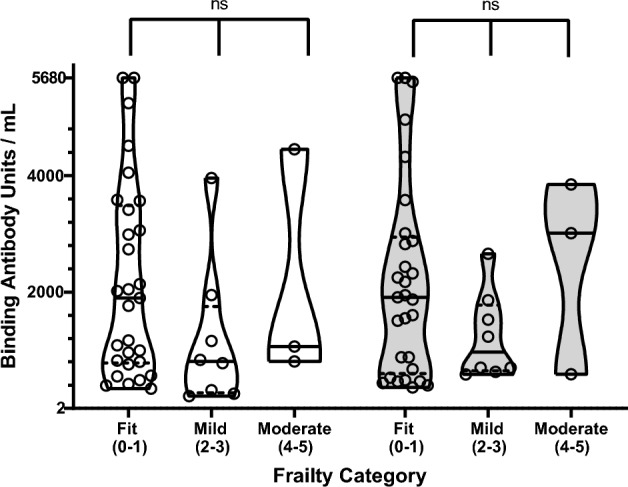
Anti-RBD IgG titers distribution according to Frailty of the whole cohort. There was no difference in antibody titers between frailty categories for all three samples (Kruskal–Wallis p = 0.648, 0.158, 0.802, respectively, for the entire cohort; p = 0.611, 0.189, 0.376, respectively, for patients who were not infected by SARS-CoV-2, Supplementary Fig. S2). Each circle represents one individual. Bars (horizontal) represent the median values, dashes represent first (lower) and third (upper) quartiles. In white the second blood sample data, in grey—the third blood sample data. ns: Kruskal–Wallis test p > 0.05. Frailty score were calculated according to the literature^13–15^.

The population studied had frailty scores from 0 to 5 with no severe frailty at all (Table 1 and Supplementary Table S1). There was no difference in anti-RBD IgG titers between frailty categories for all three samples (Kruskal–Wallis p = 0.648, 0.158, 0.802, respectively, for the entire cohort; p = 0.611, 0.189, 0.376, respectively, for the 40 patients who were not infected by COVID-19) (Fig. 2 and Supplementary Fig. S2).

### Neutralizing antibodies (nAb)

**Figure 3:**
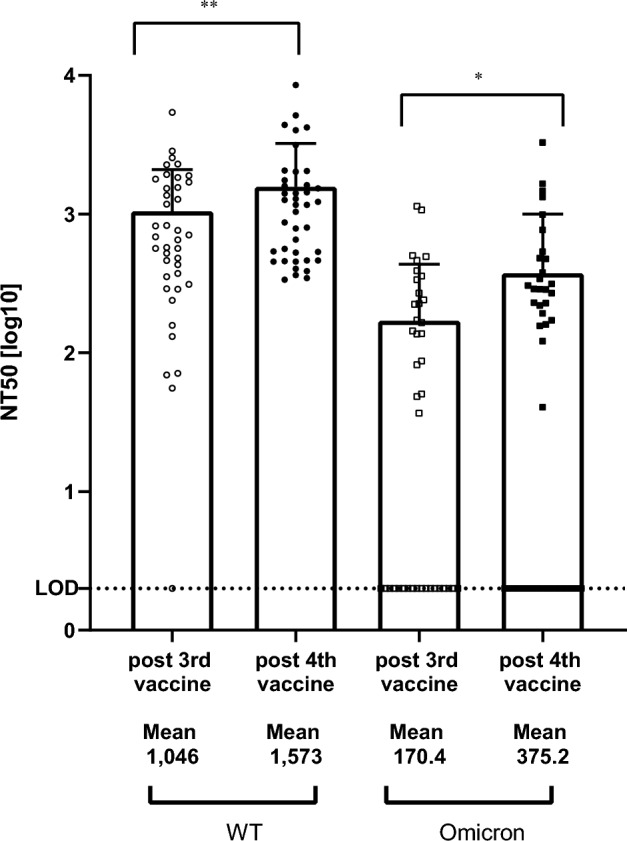
Wild type and Omicron neutralizing antibodies after the third and fourth vaccines in non-infected residents. WT: wild type SARS-CoV-2. Omicron: BA.1 variant. Neutralization capacity [NT_50_] is expressed as a function of reciprocal values of sera dilutions on a log_10_ scale. Starting at a final 1:60 dilution of the sera, the participants were assessed for both WT and Omicron neutralization antibodies by SARS-CoV-2 spike-pseudotyped VSV-GFP-ΔG reporter assay on Vero-E6 cells. Sera with NT_50_ that could not be calculated at the 1:60 dilutions were graded 2 (LOD) for graphical representations. Post 3rd vaccine: blood sampling was done at a median time of 76 days after the third vaccine. Post 4th blood: sampling was done at a median time of 99 days after the fourth vaccine. Each point corresponds to on individual. Bars represent 95%CI around the mean. **WT paired p-value = 0.0023; *Omicron paired p-value = 0.0179.

The nAb levels followed a more striking evolution. For the 40 residents not previously infected by SARS-CoV-2, if the nAb levels against the Wuhan based pseudovirus (psVWT) were low or under the LOD for the majority of the residents a week before the third dose of vaccine (data not shown), the NT_50_ titers 10–11 weeks (76 days median) after the third vaccine dose reached a median of 669 [Q1–Q3: 333; 1629] and emerged for the Omicron BA.1 based pseudovirus (psVBA1) with a median of 50 [Q1–Q3: 2; 234] (Table 2). The increase in nAb was even stronger and more statistically significant 14 weeks (99 days, median) after the fourth dose of the vaccine with a 1.7-fold increase in NT_50_ titers (median) for the psVWT versus three doses (Wilcoxon signed ranks test p = 0.001) and a fourfold increase in NT_50_ titers (median) for the psVBA.1 (Wilcoxon signed ranks test p = 0.001) (Table 2, Figs. 3).

While all the residents' sera reached NT_50_ titers over the LOD against the psVWT after the third and fourth dose of vaccine, still 14 out of 40 (versus 18 out of 40 after three vaccine doses) residents' sera were below the LOD (NT_50_ < 60) against the psVBA1, even after the fourth vaccine (Supplementary Table S1).

### Frailty and neutralizing antibodies

**Figure 4:**
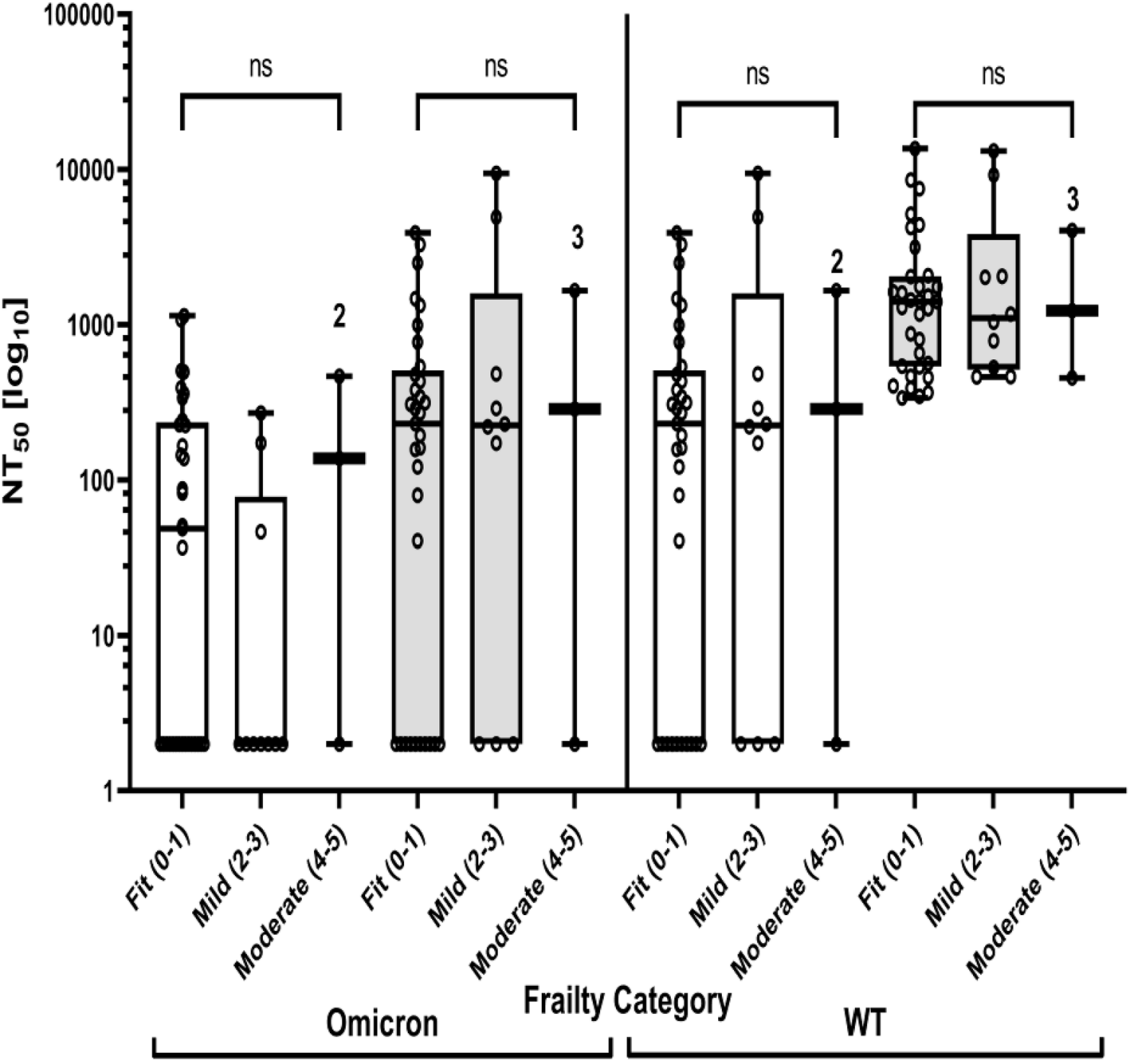
Neutralizing antibodies and frailty scores of the whole cohort. There was no difference in NT_50_ against WT between frailty categories for blood samples two (in white) or three (in grey) (Kruskal–Wallis p = 0.125, 0.975, respectively, for the entire cohort; p = 0.195, 0.863, respectively, for patients who were not infected by SARS-CoV-2, Supplementary Fig. S3). Likewise there was no difference in NT_50_ against Omicron between frailty categories for blood samples two or three (Kruskal–Wallis p = 0.279, 0.964, respectively, for the entire cohort; p = 0.245, 0.725, respectively, for patients who were not infected by SARS-CoV-2, Supplementary Fig. S3). White rectangle: second blood sampling at 76 days (median) after the third vaccine. Grey rectangle: third blood sampling after 99 days (median) after the fourth vaccine. Each circle represents one individual. Horizontal bars represent the median values, lower and upper borders of the boxes represent the first and third quartile, respectively. Whiskers represent the minimal (lower) and maximal (upper) values. ns: Kruskal–Wallis test p > 0.05. Frailty score were calculated according to the literature^13–15^.

There was no difference in NT_50_ against psVWT between frailty categories for sample 2 or 3 (Kruskal–Wallis p = 0.125, 0.975, respectively, for the entire cohort; p = 0.195, 0.863, and for patients who were not infected by COVID-19) (Fig. 4 and Supplementary Fig. S3). Likewise, there was no difference in NT_50_ against psVBA.1 between frailty categories for sample 2 or 3 (Kruskal–Wallis p = 0.279, 0.964, respectively, for the entire cohort; p = 0.245, 0.725, respectively, for patients who were not infected by COVID-19) (Fig. 4 and Supplementary Fig. S3).

### Residents who contracted COVID-19

In January first 2022, one resident was symptomatic with COVID-19 (RT-PCR test positive) but nevertheless was vaccinated with the fourth dose eight days later. Between February and April 2022, five more residents, all fourth-dose-vaccinated in January 2022, developed a laboratory confirmed COVID-19. All the vaccine escape cases of COVID-19 presented a mild to moderate disease and none required hospitalization. During that period of time, BA.1 and then BA.2 were the dominant variants. Forty residents were never found or suspected of having been exposed to SARS-CoV-2.

Fisher’s exact test found no significant difference in the frailty category between patients who did not contract COVID-19 and those who did (p = 0.747).

For the six breakthrough cases after the third vaccine dose and before the fourth dose, the mean and median anti-RBD IgG titers were slightly lower, but not significantly, than those of the 40 non-infected residents (Table 2). The median value of anti-RBD IgG levels in the six breakthrough infections, including the individual infected a week before his fourth dose of vaccine, was 2.3-fold higher and 3.3-fold higher ((Mann–Whitney U test, p = 0.002) than among the 40 residents who were never infected but were vaccinated four times (Table 2).

**Figure 5:**
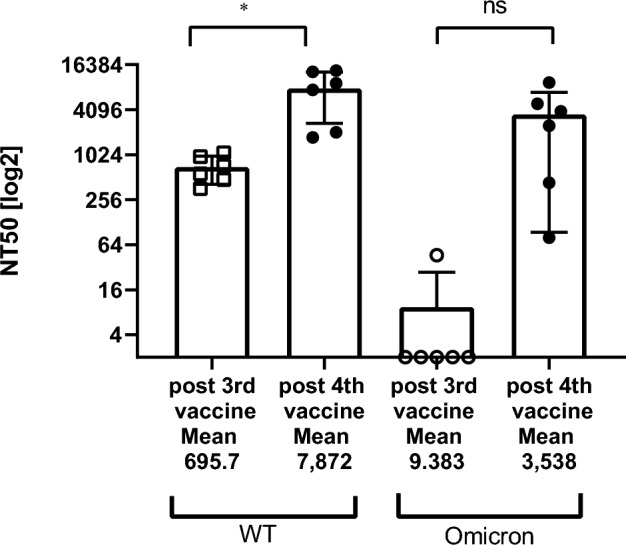
WT and Omicron neutralizing antibodies in residents before and after breakthrough infections. Serum neutralization in breakthrough infections was assessed for both wild type SARS-CoV-2 (WT) and BA.1 variant (Omicron) neutralization capacity by SARS-CoV-2 spike-pseudotyped VSV-GFP-ΔG reporter assay on Vero-E6 cells. NT5_50_ is expressed as a function of reciprocal values of sera dilutions on a log2 scale. Each circle or square represents one individual. Post 3rd vaccine: blood sampling was done at a median time of 76 days after the third vaccine. Post 4th blood: sampling was done at a median time of 99 days after the fourth vaccine. Mean values are indicated below the x-axis. *WT paired p-value = 0.0222; ns: Omicron (BA.1) paired p-value = 0.0531 but the median difference was highly significant (Wilcoxon signed ranks test p = 0.028) (Table 2).

For the six residents who had a breakthrough infection, the NT_50_ values for the third blood sample against psVWT were significantly higher than for the second sample (Wilcoxon signed ranks test p = 0.028) (Table 2). The response in nAb was even more important for the psVBA.1 with NT_50_ levels reaching close to half of the titer levels of the psVWT titers although five out of six breakthrough individuals had anti Omicron NT_50_ below the LOD prior to the breakthrough infection (Table 2, Fig. 5).

In summary, the NT_50_ titers were increased both against WT and BA.1 even eight days after the infection and persisted at least 151 days after the breakthrough infection (Table 2 and Supplementary Table S1).

As a control of potential earlier exposure to SARS-CoV-2, we measured the presence of IgG antibodies against the nucleocapsid antigen, which is not present in the vaccine formulation. The levels were below the limit of detection in all samples except for one from an individual with a breakthrough infection (data not shown).

Altogether, if looking at paired data for anti-RBD IgG, WT nAb and BA.1 nAb levels for each resident, and if we take into account the results for two pairs out of three, six individuals were down, 11 equal and 23 up. Therefore, we can consider that 58% benefited, 27% regained previous status, and 15% did not improve after the fourth dose of vaccine (Supplementary Table S1). Altogether, 85% of the residents recovered or improved their immune response after the fourth dose of vaccine, as compared to their immune status after the third dose.

## Discussion

The data presented, based on paired blood samples collected after the third and the fourth doses of the same mRNA vaccine, describe the humoral response of a well-protected elderly population (85 years, median) of whom 87% (n = 40) were never infected by SARS-CoV-2. Subsequently, as the residents were tested on a weekly basis and 40 were found free of SARS-CoV-2 during April 2020–May 2022, this study allowed the comparative evaluation of levels of anti-RBD IgG and nAb induced by the third and fourth dose of vaccine without the noise of potential COVID-19, including asymptomatic cases as described by Friedman et al.^19^.

Comparing this study to other published data is complex. The first challenge is the timing between vaccination and blood sampling that are different among the different studies^1, 3, 4, 10, 20–22^. Additionally, serological data expressed in BAU/ml are not always comparable when using different technical platforms^23^. Finally, although data obtained by nAb determination assays based on pseudovirus VSV constructs or based on live viruses are well correlated with each other, the pseudovirus lentivirus-based assays are less correlated^24^.

In the elderly population studied, the mean anti-RBD IgG levels two and a half months after the third dose and three and a half months after the fourth dose were 1987 BAU/ml (95% CI 1462; 2512) and 2025 BAU/ml (95% CI 1522; 2528) respectively and were grossly half of the values found by Eliakim-Raz et al.^20^, in a younger population (median age of 70 y [Q1–Q3: 66–74 years]) two weeks after the third and the fourth dose of the BNT162b2 vaccine, 3926 BAU/ml and 4161 BAU/ml respectively^20^. However, the increase between the third dose and the fourth dose of the mean anti-RBD IgG titers were comparable to our data, 6% and 2% respectively. These differences can be explained by the fact that the blood sampling in the study of Eliakim-Raz et al.^20^ was done two weeks after the mRNA vaccination which corresponds to the peak of the antibody response and not 10–14 weeks after vaccination as we did, which corresponds to a timing when anti-RBD IgG and nAb have decreased from their peak but reached a stable plateau over a long period of time^3, 25^. Indeed, Tut et al.^26^ showed in a study of paired samples following the third dose of an mRNA vaccine that in a median time between samples of 77 days, [73;83 ], anti-RBD titers fell by 58% from 1619 BAU/ml to 684 BAU/ml in an infection-naive group^26^*.*

Nevertheless, in another study with blood samples taken 56 days median after the third dose of an mRNA vaccine, Bruel et al.^27^ reported anti-Spike IgG levels in those without breakthrough infection of 2528.3 BAU/mL (range: 695.4–8832.0) which are similar to those we found 76 days (median) after the third dose of vaccine e.g. 1987 BAU/ml (95% CI 1462; 2512), considering the time lapse in the sampling in the two studies −20 days, and the consistently lower amount of anti-RBD IgG found when compared to anti-Spike IgG levels^27^. In addition, in the same study of Bruel et al.^27^, the data were also similar to what we found for breakthrough cases who had lower BAU values after the third vaccine dose than individuals without breakthrough infection, e.g., 1429.9 BAU/ml (670.9–3818.3) *vs* 2528.3 BAU/ml (range 695.4–8832.0)^28^ alike in our study with 1064 BAU/ml (95% CI 571; 1557) and 1987 (95% CI 1462; 2512) BAU/ml respectively (Table 2).

Even more, similar BAU levels to those reported in this study were found among a much younger population of health care workers (60.8 median age) and using the same vaccine (BNT162b2 vaccine)^1^ with levels of anti-RBD antibodies of 2102 BAU/ml four weeks after the third dose versus 1987 BAU/ml (95% CI 1462, 2512), 11 weeks after the first boost in the present study and 2684 BAU/ml (2372–3038) three weeks after the fourth dose versus 2025 BAU/ml (95% CI 1522; 2528), 14 weeks after the fourth dose in the present study.

Finally, antibody decay after the first and the second boost of mRNA vaccines have been documented in the literature^3, 17, 18, 22^. So accordingly, the anti-RBD IgG levels obtained after the second boost were significantly higher than the anti-RBD IgG levels after the first boost, when the latter were corrected for elapsed time (Table 2, Fig. 1).

Altogether infection-naïve elderly people, even at an advance age (85 median) but with no profound Frailty (Table 1), developed anti-RBD antibodies after the third dose of an mRNA vaccine at levels comparable to those found in younger populations^1, 10, 29^ and three and a half months after the fourth dose of vaccine regained or slightly improved the level of anti-RBD IgG that they had two and a half months after the third dose of vaccine (Table 2, Fig. 1).

Neutralizing antibodies have been correlated to protection against severe COVID-19 disease more than anti RBD IgG levels as shown by Gilbert et al.^30^ and by Nugent et al.^28^ but were not always assessed, especially in elderly populations, in the studies evaluating booster vaccine efficacy^5–9^.

In the previously mentioned population of health workers with a median age of 60.8 years Regev-Yochay et al.^1^, using a live virus-based assays, nAb titers of 1542 against WT and of 136.3 against BA.1 two weeks after four doses of vaccine are comparable to the titers developed by the elderly residents in this study, 1573 against WT and 375 against BA.1 (based on psVSV based assays) even taking into account the differences of methodologies^24^. Bruel et al.^27^ found that two months after the 3rd dose of vaccine in un-infected elderly population, EC50 of 1283 and 188 titers respectively for Delta and Omicron which are similar to the titers of 1046 for WT and of 169 for Omicron (Table 2) found after the third dose of vaccine in the present study. Similarly to what Bruel et al.^27^ described, we also found that the breakthrough infection cases had lower nAb after the third dose (696 and 9 for WT and Omicron respectively) (Table 2) compared to non-infected individuals and that it may be correlated to frailty scores.

In a slightly younger population (median 74 years [Q1–Q3 = 67–85 years]), 3 months after the second boost, Canaday et al.^21^ reported similar NT_50_ levels against WT (NT_50_ titers: 1333 (95% CI 931; 1908) to those found in the residents of the present study − 1573 (95% CI 1044; 2102) but the NT_50_ levels against Omicron were much higher—924 (95% CI 621; 1373) than those found in the present study—375 (95% CI 176; 575). As stated by Canaday et al.^21^ some of the volunteers in community nursing homes and veterans’ homes may have had undetected asymptomatic infection that would explain the differences found in these two studies.

Similarly, in an additional study published by Nugent et al.^28^, nursing home residents (median age 75) without prior infection had NT_50_ titers of 1272 (95% CI 746; 2170) for WT and 498 (95% CI 253; 982) for BA.1 three months post second booster, again very similar to what we found in our elderly population (Table 2). Both those without and with prior infection, had a robust geometric mean fold rise (GMFR) of 10.2 (95% CI 5.1; 20.3) and 6.5 (95% CI 4.5; 9.3) respectively in Omicron-BA.1 variant specific neutralizing antibody levels following the second booster vaccination (p < 0.001)^28^. Accordingly, we can assume a similar increase in the present study’s participants which were not sampled just before the fourth vaccine.

We can conclude that the second boost resulted for elderly residents in a significant increase of NT_50_ titers both against WT and Omicron BA.1 even 14 weeks after the fourth dose of vaccine when compared to the titers developed 11 weeks after the third dose of vaccine, in accordance with the data reported in studies that covered younger adult populations (60–75 y)^1, 21, 26, 29^.

A concern about repeated doses of vaccines is that the immune response will become exhausted by multiple boosts and thus dampened in the event of subsequent infection. While some evidences suggest that in severe cases of COVID-19, immune exhaustion can be an aggravating factor of the disease with exhausted T cell populations and therefore impeding a strong immune response as described by Jarjour et al.^31^, it has never been shown in repeated COVID-19 vaccination^32^. Moreover, in breakthrough infections after vaccine exposure “diversify of the T cell memory repertoire under current vaccination protocols continue to expand and differentiate spike-specific memory” as stated by Minervina et al.^32^. Indeed, in the six elderly residents with an Omicron breakthrough infection, the median NT_50_ values of their third blood sample against psVWT and against psVBA.1 showed a 13- and 60-fold increase (if we hypothesize that the titers were close to the LOD) respectively (Table 2) whereas for the non-infected elderly resident the fold increases were 1.7 and 4 respectively. Likewise, the mean anti-RBD IgG titers increased by 1.5-fold in non-infected residents and by 5.8 in the breakthrough cases. These data and those of the literature sustain the fact that there was no exhaustion of the immune system of the elderly residents and even more, indicate a maturation process which resulted in an improved humoral response to the Omicron BA.1 variant^22, 31, 32^. However, it must be noticed that 35% of the non-infected elderly residents did not have anti-BA.1 nAb titers over the LOD even after the fourth dose of vaccine.

The present study has some limitations as it was based on a limited number of elderly individuals, increasing the weight of individual variations that are not seen in larger studies^1, 10, 17, 18, 21, 22, 26, 27, 29, 30, 33^. However, all the residents were epidemiologically and medically followed during the study, were all sampled three times, and became their own control when comparing the humoral response (see “Methods” paragraph and Supplementary Table S1). We could not sample the elderly residents exactly a week before and three weeks after each booster to optimally evaluate their humoral response for ethical reasons: venous blood samples among elderly participants should be limited. Although the residents were tested weekly for the presence of SARS-CoV-2 during the whole period of the study, some asymptomatic cases may have been undiagnosed, which could result in some outliers^19, 21^. However, anti-nucleocapsid IgG were not found at a significant level among the residents except for one breakthrough infection individual. While anti-N antibody levels in vaccinated people is a limited marker for breakthrough infections as suggested by Dakal et al.^34^, the combination of regular antigen testing and absence of anti-nucleocapsid IgG reasonably rule out missed diagnoses. Due to the relatively small number of participants, who were mostly females, we cannot generalize our data to the entire elderly population. Yet, the present study demonstrates that the elderly have a significant humoral response after the third and fourth dose of vaccine, even when compared to younger populations^1, 10, 17, 18, 21, 22, 26, 27, 29, 30, 33^. As shown in Table Sl, seven months after the primary two doses regimen and the week before the third dose, anti-RBD levels against WT were very low and there was no point in testing nAb against the Omicron variant as the literature indicates that 80% of the individuals at 20 weeks post vaccination (two doses) had no anti-Omicron nAb^35^. We did not study the response to BA.2 or BA.5 variants, but Newman et al.^35^ showed that in adults, a third dose of BNT162b2 induced neutralizing antibody titers, providing cross-protection against Omicron BA.1 and BA.2., and Amano et al.^36^ found that after a fourth dose in an elderly population the mean NT_50_ against BA.2 and BA.5 were restored to the extent seen after the third dose. The immune cell response of the elderly residents was not investigated in the present study but Saiag et al.^22^ and Munro et al.^10^ showed significant increase in T cell response after the third and fourth vaccine dose of an mRNA vaccine. Neutralization studies were performed with a VSV based pseudovirus assay and not with live viruses but a good correlation between the two techniques has been shown in the literature^24^. Finally, we did not find any influence of frailty on anti-RBD IgG or nAb levels but this is limited by the absence of severely frail participants in the studied population. Nonetheless, another study indicated that the booster vaccination appeared to overcome the effects of frailty on spike and RBD protein antibody quantity for severely frail individuals^37^.

In conclusion, adults, including elderly adults, develop a robust immune response against the Wuhan strain and Omicron variants after the third or a fourth dose of a mRNA vaccine^10, 36, 38, 39^. The present study shows that in the Elderly (85 years median), a fourth dose maintains and even expands the functional antibodies against the ancestral Wuhan strain and the BA.1 Omicron variant. This correlates well with data on VE studies showing that the fourth dose recipients seemed to benefit an equal or even better VE when compared to third-dose recipients^5–9^. Indeed, a fourth dose of mRNA COVID-19 vaccine, provided among nursing home residents with additional protection over the first booster dose against severe COVID-19 outcomes during a time of emerging Omicron variants such BA.2, BA.2.12.1, BA.4, BA.5, B.1.1.529 and BA.2^7^.

Definitively, the fourth dose notably increased the level of neutralizing antibodies against not only the SARS-CoV-2 wild type but also against the variant BA.1 (Fig. 3) in COVID-19 naïve elders, and vaccine escape infections resulted in enhanced immune response (Table 2, Fig. 5), supporting the fact that the immune system of these elders did not show signs of exhaustion.

As the Elderly are the most at-risk population, their ability to respond to four vaccine doses delivered within a year speaks to a robustness of immune response, rather than exhaustion. As such, part of a multifaceted response to the COVID-19 pandemic should continue to include booster schedules. There is a definitive need for new and better vaccine compositions that would include new antigens beside the spike antigen and antigens shared by many variants providing a wider protection for emerging and future variants.

## Methods

### Study design and participants

This study was performed within a cohort of elderly residents living in an assisted living facility where there were no confirmed COVID cases among residents at time of recruitment (May–August 2021). Participants were interviewed to complete a structured questionnaire, which included sociodemographic characteristics, current health condition, a measurement of frailty and known exposures to COVID-19 and subsequent tests and infections^13–15^. Three blood samples were collected between August 2021 and May 2022. The first blood sample was taken at a median of 194 days after the second vaccine dose (and a week before the third vaccine dose), the second blood sample was taken at a median of 76 days after the third vaccine dose, and the third blood sample was taken at a median of 99 days after the fourth vaccine dose (Supplementary Fig. S1). Within the framework of the MoH "Defense for Our Parents" Guidelines^16^, all participants were regularly screened for COVID-19, using either Real-Time Polymerase Chain Reaction (RT-PCR) or antigen tests. People who were immunocompromised were excluded from the cohort study.

The assisted living facility was without known COVID-19 cases at time of recruitment but cases were detected starting January 2022, during the Omicron wave. Among cohort study participants who contributed all three blood samples, six participants were diagnosed with COVID-19 between January and April 2022, one participant a week before the fourth dose and five after the fourth dose (Supplementary Table S1). All the vaccine escape cases of COVID-19 presented a mild to moderate disease and none required hospitalization. The remaining 40 participants were not diagnosed with COVID-19, confirmed by weekly antigen tests. It is important to note that this private institution has permanent medical staff (physician and nurses) dedicated to the elderly and is composed of private units (studio apartments), which facilitated residents’ ability to isolate throughout the pandemic response.

Our research protocol was approved by the National Committee for Human Medical Research (The Israeli Minister of Health-MOH) in the office of the Chief Scientist (CoR-MOH-058-2020) and performed in accordance with the Declaration of Helsinki. Informed consent was obtained from all participants and/or their legal guardians. The study was performed in compliance with the provisions of the Declaration of Helsinki from the World Medical Association and good clinical practice (GCP) guidelines. All methods were performed in accordance with the relevant guidelines and regulations.

### Serology

Blood samples were collected in VACUETTE 8 ml CAT Serum Separator Clot Activator tubes and after coagulation at room temperature, the tubes were centrifuged (21 °C, 3500*g*, 10 min). Sera were aliquoted and immediately frozen at − 74 °C. After one cycle of freeze-thawing, sera were tested for immunoglobulin G (IgG) antibodies against the SARS-CoV-2 spike RBD using the commercial Chemiluminescent Microparticle ImmunoAssay (CMIA) SARS-CoV-2 IgG II Quant (Abbott, IL, USA), according to the manufacturer's instructions at the certified laboratory of the Hebrew University medical school affiliated Shaare Zedek Medical Center (Jerusalem, Israel). The antibody titers were obtained in arbitrary antibody units (AU/ml) and were converted by multiplying them by a factor of 0.142 (according to the manufacturer’s instructions) to Binding Antibody Unit (BAU/ml), adapted to the WHO standard for SARS-CoV-2 immunoglobulin^33^. Anti-Nucleocapsid (N) IgG antibodies were evaluated in a made-in-house Enzyme-Linked Immunosorbent Assay (ELISA) test, as described in detail by Stolovich-Rain et al.^40^.

### Neutralizing antibodies

Neutralization assay was performed using propagation—incompetent, single-round infectious particles, i.e.—vesicular stomatitis virus spike SARS-CoV-2 pseudovirus (psV) with GFP-reporter^40^, pseudotyped with the historical Wuhan (psVWT) or the BA.1 sequence (psVBA.1) that were constructed as described by Stolovich-Rain et al.^40^. Neutralization assays based on psSARS-CoV-2 have been shown to be highly correlative to authentic SARS-CoV-2 virus micro-neutralization assay^40^. Following titration, 100–300 focus forming units (FFU) of psSARS-2 were incubated with two to threefold serial dilutions of heat inactivated (56 °C for 30 min) tested sera. After incubation for 60 min at 37 °C, virus/serum mixture was transferred to Vero E6 cells grown to 75–80% confluence in 96-well plates. The plates were incubated for 18–22 h, and 50% focus reduction titer (NT_50_) was calculated by counting green fluorescent foci using an automated fluorescence microscope (Cytation5, Agilent, BioTek)^24, 41^. NT_50_ was calculated using the method established by Reed and Muench^42^. Sera not capable of reducing viral replication by 50% at a final dilution of 1:60 were considered non-neutralizing; limit of detection (LOD) = 60. This value is two to three times-fold higher than the value corresponding to a level of protection against infection or serious disease as described by Khoury et al.^25^. For a clear graphical presentation, samples below the LOD were marked as a titer of two^42^.

### Statistical analysis

Descriptive statistics were presented as frequencies and percentages for categorical variables and as median values with interquartile range or mean values with standard deviations for continuous variables, as appropriate. 95% Confidence Intervals (CIs) were presented when performing a graphical comparison between groups. Non-parametric tests were used when applicable. All data analyses were performed with the IBM SPSS (version 22). Plots were created using SPSS and GraphPad Prism 9.0 (GraphPad Software, Inc, San Diego, CA). Statistical tests were two-tailed and an alpha value of 0.05 was considered as the cutoff for statistical significance.

Antibody count data was censored above 5,680 BAU/ml and values < 2 were censored for NT_50_ data.

The first sample was taken after the second vaccine. The second sample was taken after the third vaccine. The third sample was taken after the fourth vaccine.

### Supplementary Information

Supplementary Information.

## Data Availability

All data produced in the present study are included in the Supplementary Information.

## References

[1] Regev-Yochay G (2022). Efficacy of a fourth dose of COVID-19 mRNA vaccine against Omicron. N. Engl. J. Med..

[2] Azim Majumder MA, Razzaque MS (2022). Repeated vaccination and “vaccine exhaustion”: Relevance to the COVID-19 crisis. Expert Rev. Vaccines.

[3] Canetti M (2022). Six-month follow-up after a fourth BNT162b2 vaccine dose. N. Engl. J. Med..

[4] Anderson EM (2022). SARS-CoV-2 infections elicit higher levels of original antigenic sin antibodies compared with SARS-CoV-2 mRNA vaccinations. Cell Rep..

[5] Nordström P, Ballin M, Nordström A (2022). Effectiveness of a fourth dose of mRNA COVID-19 vaccine against all-cause mortality in long-term care facility residents and in the oldest old: A nationwide, retrospective cohort study in Sweden. Lancet Reg. Health Eur..

[6] Fabiani M (2023). Relative effectiveness of a 2nd booster dose of COVID-19 mRNA vaccine up to four months post administration in individuals aged 80 years or more in Italy: A retrospective matched cohort study. Vaccine.

[7] McConeghy KW (2022). Effectiveness of a second COVID-19 vaccine booster dose against infection, hospitalization, or death among nursing home residents—19 states, March 29–July 25, 2022. MMWR Morb. Mortal Wkly. Rep..

[8] Muhsen K (2022). Association of receipt of the fourth BNT162b2 dose with Omicron infection and COVID-19 hospitalizations among residents of long-term care facilities. JAMA Intern. Med..

[9] Grewal R (2022). Effectiveness and duration of protection of a fourth dose of COVID-19 mRNA vaccine among long-term care residents in Ontario, Canada. J. Infect. Dis..

[10] Munro APS (2022). Safety, immunogenicity, and reactogenicity of BNT162b2 and mRNA-1273 COVID-19 vaccines given as fourth-dose boosters following two doses of ChAdOx1 nCoV-19 or BNT162b2 and a third dose of BNT162b2 (COV-BOOST): A multicentre, blinded, phase 2, randomised trial. Lancet Infect. Dis..

[11] Anonymous. https://www.afro.who.int/news/over-two-thirds-africans-exposed-virus-which-causes-covid-19-who-study; https://www.afro.who.int/news/over-two-thirds-africans-exposed-virus-which-causes-covid-19-who-study.

[12] Levin AT, Jylhävä J, Religa D, Shallcross L (2022). COVID-19 prevalence and mortality in longer-term care facilities. Eur. J. Epidemiol..

[13] Rockwood K (2005). A global clinical measure of fitness and frailty in elderly people. CMAJ.

[14] COVID-19 Cumulative Infection Collaborators (2022). Estimating global, regional, and national daily and cumulative infections with SARS-CoV-2 through Nov 14, 2021: A statistical analysis. Lancet.

[15] Raîche M, Hébert R, Dubois M-F (2008). PRISMA-7: A case-finding tool to identify older adults with moderate to severe disabilities. Arch. Gerontol. Geriatr..

[16] Tsadok-Rosenbluth S, Hovav B, Horowitz G, Brammli-Greenberg S (2021). Centralized management of the COVID-19 pandemic in long-term care facilities in Israel. J. Long Term Care.

[17] Gilboa M (2022). Durability of immune response after COVID-19 booster vaccination and association with COVID-19 omicron infection. JAMA Netw. Open.

[18] Grassi T (2022). Kinetics of humoral immunity against SARS-CoV-2 in healthcare workers after the third dose of BNT162b2 mRNA vaccine. Vaccines.

[19] Friedman SM (2022). Antibody seroprevalence, infection and surveillance for SARS-CoV-2 in residents and staff of New Jersey long-term care facilities. J. Community Health.

[20] Eliakim-Raz N (2022). Antibody titers after a third and fourth SARS-CoV-2 BNT162b2 vaccine dose in older adults. JAMA Netw. Open.

[21] Canaday DH (2023). SARS-CoV-2 antibody responses to the ancestral SARS-CoV-2 strain and omicron BA.1 and BA.4/BA.5 variants in nursing home residents after receipt of bivalent COVID-19 vaccine—Ohio and Rhode Island, September–November 2022. MMWR Morb. Mortal Wkly. Rep..

[22] Saiag E, Alcalay Y, Marudi O, Orr-Urtreger A, Hagin D (2023). Cellular and humoral immune response to the fourth Pfizer-BioNTech COVID-19 vaccine dose in individuals aged 60 years and older. Vaccine.

[23] Perkmann T (2023). Comparison of five Anti-SARS-CoV-2 antibody assays across three doses of BNT162b2 reveals insufficient standardization of SARS-CoV-2 serology. J. Clin. Virol..

[24] Riepler L (2020). Comparison of four SARS-CoV-2 neutralization assays. Vaccines.

[25] Khoury DS (2021). Neutralizing antibody levels are highly predictive of immune protection from symptomatic SARS-CoV-2 infection. Nat. Med..

[26] Tut G (2023). Strong peak immunogenicity but rapid antibody waning following third vaccine dose in older residents of care homes. Nat. Aging.

[27] Bruel T (2022). Neutralising antibody responses to SARS-CoV-2 omicron among elderly nursing home residents following a booster dose of BNT162b2 vaccine: A community-based, prospective, longitudinal cohort study. EClinicalMedicine.

[28] Nugent C (2023). Second monovalent SARS-CoV-2 mRNA booster restores Omicron-specific neutralizing activity in both nursing home residents and health care workers. Vaccine.

[29] Salvagno GL (2021). Anti-spike S1 IgA, anti-spike trimeric IgG, and anti-spike RBD IgG response after BNT162b2 COVID-19 mRNA vaccination in healthcare workers. J. Med. Biochem..

[30] Gilbert PB (2022). A Covid-19 milestone attained—A correlate of protection for vaccines. N. Engl. J. Med..

[31] Jarjour NN, Masopust D, Jameson SC (2021). T cell memory: Understanding COVID-19. Immunity.

[32] Minervina AA (2022). SARS-CoV-2 antigen exposure history shapes phenotypes and specificity of memory CD8+ T cells. Nat. Immunol..

[33] Gallais F (2021). Evolution of antibody responses up to 13 months after SARS-CoV-2 infection and risk of reinfection. EBioMedicine.

[34] Dhakal S (2023). Reconsideration of antinucleocapsid IgG antibody as a marker of SARS-CoV-2 infection postvaccination for mild COVID-19 patients. Open Forum Infect. Dis..

[35] Newman J (2022). Neutralizing antibody activity against 21 SARS-CoV-2 variants in older adults vaccinated with BNT162b2. Nat. Microbiol..

[36] Amano M (2022). Restoration of neutralization activity against Omicron BA.2 and BA.5 in older adults and individuals with risk factors following the fourth dose of severe acute respiratory syndrome coronavirus 2 BNT162b2 vaccine. J. Infect. Dis..

[37] Semelka CT (2023). Frailty impacts immune responses to Moderna COVID-19 mRNA vaccine in older adults. Immun. Ageing.

[38] Painter MM (2023). Prior vaccination enhances immune responses during SARS-CoV-2 breakthrough infection with early activation of memory T cells followed by production of potent neutralizing antibodies. BioRxiv.

[39] Renia L (2022). Lower vaccine-acquired immunity in the elderly population following two-dose BNT162b2 vaccination is alleviated by a third vaccine dose. Nat. Commun..

[40] Stolovich-Rain M (2022). Intramuscular mRNA BNT162b2 vaccine against SARS-CoV-2 induces neutralizing salivary IgA. Front. Immunol..

[41] Whitt MA (2010). Generation of VSV pseudotypes using recombinant ΔG-VSV for studies on virus entry, identification of entry inhibitors, and immune responses to vaccines. J. Virol. Methods.

[42] Reed LJ, Muench H (1938). A simple method of estimating fifty per cent endpoints. Am. J. Epidemiol..

