# Supplementary material for: Antibody response in elderly vaccinated four times with an mRNA anti-COVID-19 vaccine
Source: Sci Rep. 2023 Aug 29;13:14165. doi: 10.1038/s41598-023-41399-5 (PMC10465611; doi:10.1038/s41598-023-41399-5)
Supplement: Supplementary file 1 — Supplementary Information. [file 41598_2023_41399_MOESM1_ESM.pdf]

Supplementary Information

Antibody Response in Elderly Vaccinated Four Times with an mRNA Anti-COVID-19 Vaccine

Alexander Rouvinski[1#*], Ahuva Friedman[1#], Saveliy Kirillov[1,2#], Jordan Hannink Attal[3,4], Sujata Kumari[1,5], Jamal Fahoum[5] , Reuven Wiener [5], Sophie Magen[6], Yevgeni Plotkin[7], Daniel Chemtob[b,f] , Herve Bercovier[a].

[1]Department of Microbiology and Molecular Genetics, Faculty of Medicine, Hebrew University of Jerusalem, Israel

[2]Department of General Biology and Genomics, L.N. Gumilyov Eurasian National University, Astana, Kazakhstan

[3]Braun School of Public Health and Community Medicine, Faculty of Medicine, Hebrew University of Jerusalem, Israel

[4]Department of Tuberculosis and AIDS, State of Israel Ministry of Health, Jerusalem, Israel

[5]Department of Biochemistry and Molecular Biology, Faculty of Medicine, Hebrew University of Jerusalem, Israel

[6]Department of Clinical Biochemistry, Shaare Zedek Medical Center, Jerusalem, Israel

[7]Department of Anesthesiology, Critical Care and Pain Medicine, Hadassah Medical Center and Faculty of Medicine, Hebrew University of Jerusalem, Israel

*****Corresponding Author: alexander.rouvinski@mail.huji.ac.il

[#]Equal participation

Figure S1 Time line of sampling and vaccination

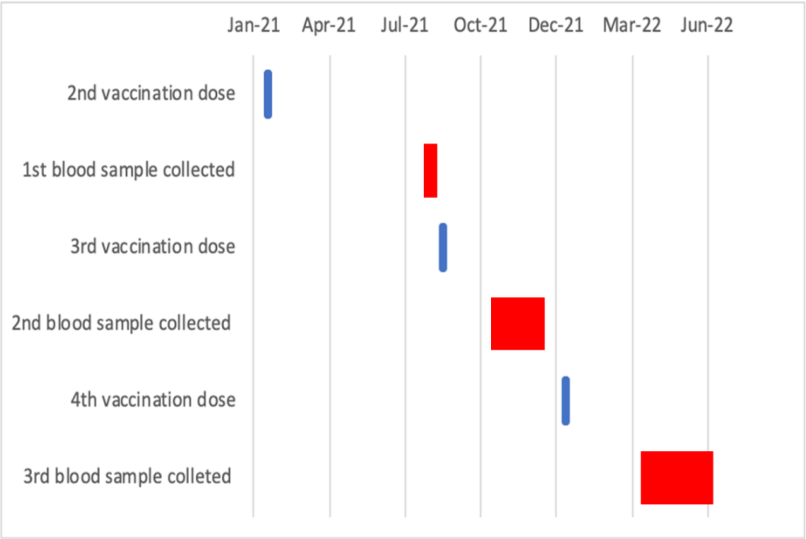

Legends of Figure S1.

In red, blood sampling, in blue vaccination time. The bar for January 2021 represents the time of the second vaccine, three weeks after the first vaccine.

Figure S2 Anti-RBD IgG titers distribution according to Frailty in non-infected residents

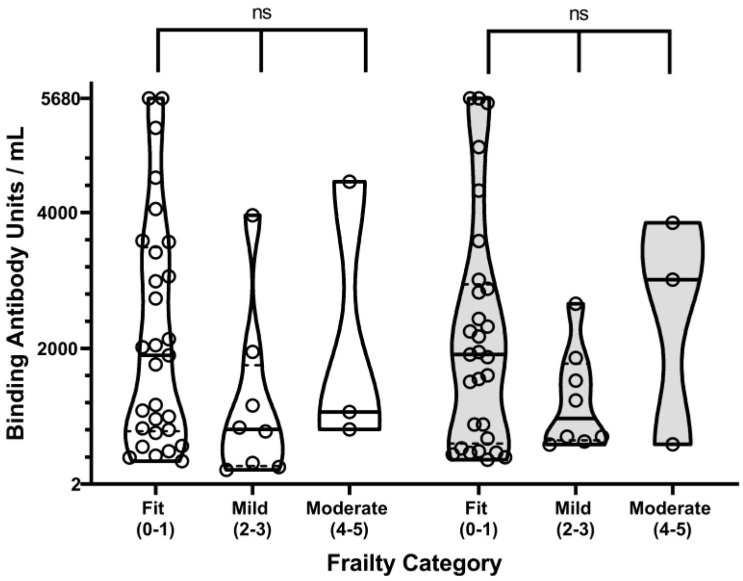

Legends of Figure S2. **Anti-RBD IgG titers distribution according to Frailty in non-infected residents**

Each circle represents one individual. In white the second blood sample data, in grey the third blood sample data. The horizontal bars represent the median anti-RBD IgG titer in BAU/ml. There was no difference in antibody titer between frailty categories for all three samples (Kruskal-Wallis p=0.611, 0.189, 0.376, respectively, for the 40 patients who were not infected by SARS-CoV-2). Frailty score were calculated according to Rockwood et al. [13].

Figure S3 . Neutralizing antibodies levels and frailty in non-infected residents

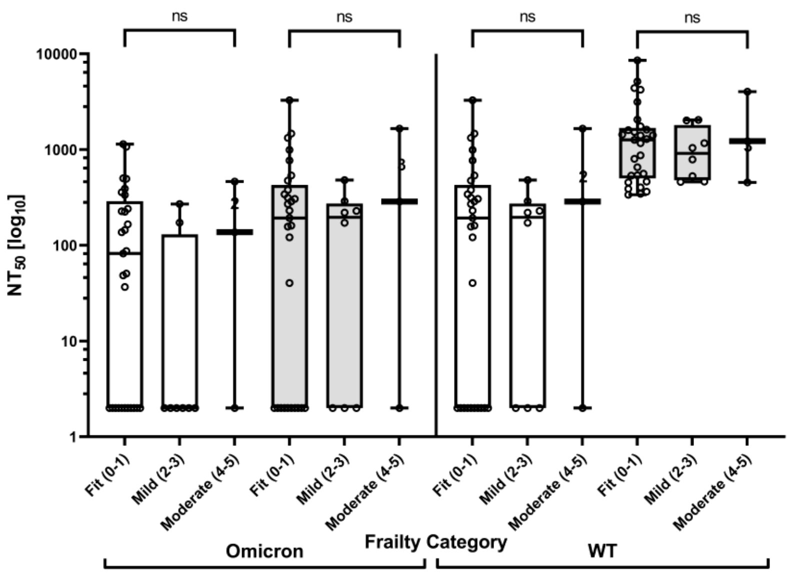

Legend of Figure S3. **Neutralizing antibodies levels and frailty in non-infected residents**

There was no difference in NT_{50} against Omicron (Kruskal-Wallis; p=0.245) or WT (Kruskal-Wallis; p=0.725) between frailty categories for sample 2 or 3 for residents who were not infected by SARS-CoV-2. Each circle represents one individual. White rectangle: second blood sampling at 76 days (median) after the third vaccine. Grey rectangle: third blood sampling after 99 days (median) after the fourth vaccine. Horizontal bars represent the median values. Whisker bars the minimal and maximal values (IQR: quarter 1 and quarter 4). ns: Kruskal-Wallis; p>0.05. Frailty score were calculated according to Rockwood et al. [13].

Table S1 Characteristics and humoral response of the population studied

			Anti-RBD IgG Antibody Titer (BAU/ml)			NT_{50}				Days Elapsed between										
**Resident Code**	**Age**	**BT**	**Sample 1**	**Sample 2**	**Sample 3**	**Sample 2**		**Sample 3**		**2nd Vac. and Sample 1**	**3rd Vac. and Sample 2**	**4th Vac. and Sample 3**	**BT and Sample 3**	**FS**	**Sex**	**DM**	**HTN**	**Smoking**	**Obesity**	**Frailty Category**
						Omicron	WT	Omicron	WT											
2001	78	0	50	835	1859	2	131	2	787	194	121	97	NR	2	1	No	No	No	No	Mild (2-3)
2004	78	0	12	340	462	2	69	2	464	194	70	143	NR	0	1	No	Yes	Yes	No	Fit (0-1)
2005	78	0	88	563	439	2	482	2	538	194	81	99	NR	0	0	No	No	Yes	No	Fit (0-1)
2007	14	0	44	807	586	2	374	2	454	194	70	99	NR	4	1	No	Yes	No	No	Moderate (4-5)
2009	76	0	126	1897	2878	166	1535	379	1595	194	70	99	NR	1	1	No	Yes	No	No	Fit (0-1)
2010	87	0	66	4051	2823	82	652	2	1166	194	70	99	NR	0	1	No	Yes	No	No	Fit (0-1)
2012	93	0	11	210	629	2	71	2	462	194	70	135	NR	3	1	No	No	Yes	No	Mild (2-3)
2013	96	0	25	776	700	2	354	289	527	194	70	135	NR	2	1	No	No	No	No	Mild (2-3)
2014	94	0	23	252	585	2	158	220	459	194	121	99	NR	2	1	Yes,	Yes	Yes	No	Mild (2-3)
2017	86	0	33	311	2657	2	56	172	2045	194	70	99	NR	2	0	No	No	No	No	Mild (2-3)
2018	93	0	21	800	526	2	565	2	455	194	81	99	NR	0	1	No	No	No	Yes	Fit (0-1)
2019	88	0	25	2016	879	502	1278	536	535	194	81	135	NR	0	1	No	No	No	No	Fit (0-1)
2020	85	0	107	4513	2249	358	1698	342	1412	194	81	135	NR	0	0	No	Yes	No	No	Fit (0-1)
2021	79	0	368	2136	2433	495	822	771	1406	194	81	135	NR	0	1	No	No	No	No	Fit (0-1)
2022	84	0	360	2988	4323	137	1788	287	1751	194	81	99	NR	1	1	No	Yes	No	No	Fit (0-1)
2023	86	0	17	5680	5680	1071	2843	1469	3147	194	70	99	NR	0	1	No	No	No	No	Fit (0-1)
2024	88	0	26	757	673	2	239	2	337	194	70	99	NR	0	0	Yes,	No	Yes	No	Fit (0-1)
2025	91	0	240	485	360	2	289	2	364	194	70	135	NR	0	1	No	Yes	No	No	Fit (0-1)
2026	85	0	519	5680	5680	1138	2268	993	4394	194	81	99	NR	1	1	No	Yes	No	Yes	Fit (0-1)
2027	91	0	383	5245	4963	144	5423	270	8535	194	81	135	NR	1	1	No	Yes	Yes	No	Fit (0-1)
2028	83	0	8	994	486	2	566	2	388	195	81	99	NR	0	1	No	Yes	Yes	Yes	Fit (0-1)
2030	91	0	147	3565	1944	336	2546	314	1437	195	70	99	NR	0	0	No	No	No	No	Fit (0-1)
2032	78	0	19	2734	1869	49	1559	2	1616	195	70	99	NR	1	1	No	No	No	No	Fit (0-1)
2033	83	0	46	959	1507	2	575	2	1266	195	81	99	NR	0	1	No	No	No	No	Fit (0-1)
2034	78	0	400	1159	699	2	706	2	1039	195	70	99	NR	3	1	No	Yes	No	No	Mild (2-3)
2035	75	0	220	3582	2174	241	1929	157	800	195	81	143	NR	0	1	No	Yes	No	Yes	Fit (0-1)
2037	99	0	105	1947	1234	173	1365	229	1165	195	70	135	NR	3	1	No	Yes	No	No	Mild (2-3)
2038	81	0	38	1763	1602	87	685	160	1290	195	70	99	NR	0	1	No	Yes	No	Yes	Fit (0-1)
2039	85	0	34	822	464	227	433	121	403	195	84	143	NR	0	1	No	Yes	No	No	Fit (0-1)
2040	86	0	80	3415	1908	224	829	306	872	195	81	143	NR	0	1	No	No	No	No	Fit (0-1)
2041	82	0	127	1169	3581	2	763	1327	4221	195	81	99	NR	1	1	No	Yes	No	No	Fit (0-1)
2043	88	0	71	4455	3850	464	1897	1655	4029	195	70	99	NR	5	1	No	Yes	No	Yes	Moderate (4-5)
2045	92	0	16	551	5614	2	312	475	1530	195	70	135	NR	0	1	No	Yes	Yes	No	Fit (0-1)
2046	82	0	86	1084	877	37	463	40	561	195	70	99	NR	0	1	No	No	No	No	Fit (0-1)
2048	93	0	42	420	1548	2	2	230	655	195	81	99	NR	1	1	No	No	No	No	Fit (0-1)
2049	83	0	75	3960	1524	270	1834	481	2019	195	70	135	NR	2	1	No		No	No	Mild (2-3)
2050	87	0	11	393	395	2	288	2	345	195	70	135	NR	0	0	No	No	No	No	Fit (0-1)
2051	87	0	151	3059	3008	391	2299	3277	5151	196	84	99	NR	1	1	No	Yes	No	Yes	Fit (0-1)
2052	86	0	60	2047	2320	51	1180	193	2061	196	81	143	NR	1	1	No	Yes	Yes	No	Fit (0-1)
2053	83	0	14	1063	3012	137	523	286	1226	196	84	99	NR	4	1	Yes	No	Yes	Yes	Moderate (4-5)
2003	88	1	37	1037	1921	2	723	431	1743	194	81	135	14	0	1	No	No	No	No	Fit (0-1)
2006	80	1	32	934	5680	2	567	4907	13132	194	81	143	47	2	1	No	No	No	No	Mild (2-3)
2011	82	1	11	210	5680	2	476	3890	13624	194	81	143	111	1	1	No	Ye	No	No	Fit (0-1)
2015	80	1	51	1572	2979	2	1089	80	2036	194	70	99	8	1	1	Yes,	Y	Yes	Yes	Fit (0-1)
2016	90	1	85	698	5680	46	360	9422	9190	194	70	143	151	2	1	No	No	No	No	Mild (2-3)
2047	82	1	140	1935	5680	2	959	2499	7508	195	70	99	50	1	1	No	No	No	No	Fit (0-1)

Legends to Table S1. **Characteristics and humoral response of the population studied**

BT: breakthrough infection (BA.1), 0= none; 1= yes. NR=non relevant. Vac.: Vaccine.

Sample1 is the blood sampling one week before the first boost (third vaccine). Sample 2

is the blood sampling 76 days (median) after the first boost. Sample 3 is the blood sampling 99 days (median) after the second boost (fourth vaccine). FS: Frailty score were calculated according to Rockwood et al. [13]. Sex: 0 is Female, 1 is Male. DM: Diabetes mellitus, No is for no Diabetes, Yes for the presence of Diabetes balanced except for resident 2053 who did not balance his Diabetes. HTN: Hypertension, No is for no hypertension, Yes is for the presence of hypertension controlled by drugs. Anti-RBD IgG were measured in samples taken one week before the third vaccine,76 and 99 days (median) after third (post third vaccine) and fourth (post fourth vaccine) vaccines, by Chemiluminescent Microparticle ImmunoAssay (CMIA) SARS-CoV-2 IgG II Quant (Abbott, IL, USA), and expressed as binding antibody units (BAU) per ml. NT_{50}: serum neutralization by the sera (starting at a 1/60 dilution) of the participants was assessed for both WT and Omicron by SARS-CoV-2 spike-pseudotyped VSV-GFP-ΔG reporter assay on Vero-E6 cells. Neutralization capacity [NT_{50}] is expressed as a function of reciprocal values of sera dilutions. Sera with NT_{50} that could not be calculated at the 1/60 dilution were graded 2 for graphical representations. WT: wild type SARS-CoV-2. Omicron: BA.1 SARS-CoV-2.
